# RNF216 contributes to proliferation and migration of colorectal cancer via suppressing BECN1-dependent autophagy

**DOI:** 10.18632/oncotarget.9433

**Published:** 2016-05-18

**Authors:** Hui Wang, Yanan Wang, Liu Qian, Xue Wang, Hailiang Gu, Xiaoqiang Dong, Shiqian Huang, Min Jin, Hailiang Ge, Congfeng Xu, Yanyun Zhang

**Affiliations:** ^1^ Shanghai Institute of Immunology, Institutes of Medical Sciences, Shanghai Jiao Tong University School of Medicine (SJTUSM) and Key Laboratory of Stem Cell Biology, Institute of Health Sciences, Shanghai Institutes for Biological Sciences, Chinese Academy of Sciences & SJTUSM, Shanghai, China; ^2^ Department of General Surgery, The First Affiliated Hospital of Soochow University, Suzhou, China

**Keywords:** RNF216, BECN1, autophagy, colorectal cancer

## Abstract

Originally identified as an E3 ligase regulating toll-like receptor (TLR) signaling, ring finger protein 216 (RNF216) also plays an essential role in autophagy, which is fundamental to cellular homeostasis. Autophagy dysfunction leads to an array of pathological events, including tumor formation. In this study, we found that RNF216 was upregulated in human colorectal cancer (CRC) tissues and cell lines, and was associated with progression of CRC. RNF216 promoted CRC cell proliferation and migration *in vitro* and *in vivo*, largely by enhancing proteasomal degradation of BECN1, a key autophagy regulator and tumor suppressor. RNF216 restricted CRC cell autophagy through BECN1 inhibition under nutritional starvation conditions. RNF216 knockdown increased the autophagy, limiting CRC cell proliferation and migration. Moreover, *BECN1* knockdown or autophagy inhibition restored proliferation and migration of *RNF216*-knockdown CRC cells. Collectively, our results suggested that RNF216 promoted CRC cell proliferation and migration by negatively regulating BECN1-dependent autophagy. This makes RNF216 as a potential biomarker and novel therapeutic target for inhibiting CRC development and progression.

## INTRODUCTION

Globally, colorectal cancer (CRC) is one of the most common cancers with high incidence and cancer associated mortality [1, 2]. Even in areas historically at low risk, such as China, Japan and Korea, CRC incidence is rapidly increasing in recent years [1, 3, 4]. However, CRC patient prognosis has not significantly improved, especially in cases of advanced disease. Factors predisposing patients to CRC include heredity, chronic inflammation, dietary patterns, obesity and smoking. CRC molecular mechanisms are complex, involving gene mutations, epigenetic modifications, aberrant protein ubiquitination and signaling crosstalk. Clarifying the mechanisms of CRC carcinogenesis and development will contribute to enhanced patient diagnosis and treatment.

Autophagy, a conserved mechanism maintaining cellular homeostasis, contributes to both normal and pathological processes. Improper autophagy has been associated with various diseases, including cancer [5–8]. Autophagy and autophagy-related genes are over or downregulated in cancers, which are both significantly associated with poor prognosis, suggesting the complicated biological role of autophagy in cancer [9–13]. The currently accepted hypothesis is that autophagy plays dual and contradictory roles in CRC and this appears context dependent. However, the precise mechanisms leading to CRC cell autophagy activation or inhibition are still unclear [14–17]. So exploring the mechanisms of autophagy participating in CRC and controlling autophagy properly will benefit diagnosis and current therapies of CRC.

Ring finger protein 216 (RNF216), an E3 ubiquitin-protein ligase, mediates ubiquitination and proteolytic degradation of several toll-like receptor (TLR), such as TLR4 and TLR9, restricting TLR signaling intensity and duration [18]. RNF216 can ubiquitinate the downstream molecules of TLRs and tumor necrosis factor (TNF) receptor 1, such as receptor interacting serine/threonine-protein kinase 1 (RIP1), negatively regulating TNF-α signaling [19]. RNF216 also targets tumor necrosis factor receptor-associated factor 3 (TRAF3) for degradation, regulating RNA virus infection [20], and interacts with BECN1 in macrophages, inhibiting autophagy and impacting macrophage response to infection [21]. Thus, RNF216 plays an important role in regulating innate immunity, RNA virus infection and autophagy, all of which are closely related to CRC tumorigenesis. Yet, the role of RNF216 in the tumorigenesis and progression of CRC has been rarely clarified.

In this study, we found that RNF216 is significantly upregulated in human CRC tissues and cell lines, and is associated with disease progression. *RNF216* knockdown inhibited CRC cell proliferation and migration by promoting BECN1-dependent autophagy under starvation conditions. Our work provides a potential therapeutic strategy for inhibiting CRC development and progression by targeting RNF216.

## RESULTS

### RNF216 is upregulated in colorectal cancer

**Figure 1:**
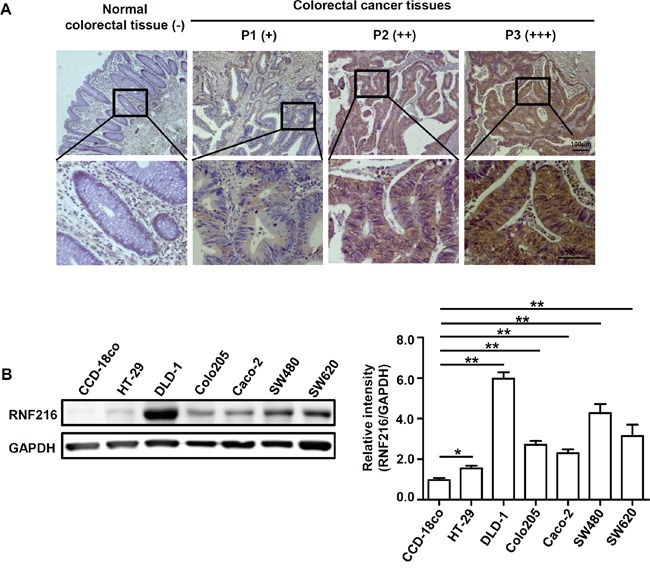
RNF216 expression is upregulated in human CRC tissues and cell lines **A.** Human normal colorectal tissues and CRC tissues were sectioned and subjected to IHC staining against RNF216. Scale bar = 100μm (top), Scale bar = 50μm (bottom). Representative images are shown. **B.** CCD-18co, HT-29, DLD-1, Colo205, Caco-2, SW480 and SW620 cells were lysed and RNF216 was detected via immunoblotting. GAPDH was used as loading control. Bands were quantified using densitometry with three independent experiments. Data is shown as mean ± SEM (*P<0.05, **P<0.001).

Immunohistochemical (IHC) staining and immunoblotting analysis were used to measure RNF216 expression in 86 human CRC tissues and 7 cell lines. Table 1 shows patient clinical features and statistical analyses of IHC staining. RNF216 exhibited positive cytoplasmic staining (Figure 1A); staining was greater in cancerous lesions compared to adjacent noncancerous lesions (*P*<0.001). RNF216 expression correlated with advanced clinical stage (*P*<0.001) and positive lymph node metastasis (*P*<0.001), suggesting a correlation between RNF216 expression and CRC progression (Table 1). RNF216 expression was also higher in all six CRC cell lines tested, compared with the normal colon epithelial cell line, CCD-18co (Figure 1B). We used DLD-1 and SW480 cells, two representative cell lines with high RNF216 expression, for further studies.

**Table 1:** Correlation of RNF216 expression with clinicopathologic features in CRC

Clinicopathologic parameters	Case No.	RNF216 expression		*P* value
		Low	High	
**Total cases**	86	45	41	
**Tissue type**				***P* < 0.001**
Normal colorectal tissue	86	86	0	
CRC tissue	86	45	41	
**Age**				***P* = 0.890**
≤ 60 years	35	18	17	
> 60 years	51	27	24	
**Gender**				***P* = 0.084**
Male	43	18	25	
Female	43	27	16	
**Tumor size**				***P* = 0.665**
≤ 5 cm	36	20	16	
> 5 cm	41	25	25	
**TNM stage**				***P* = 0.019**
I	12	9	3	
II	28	19	9	
III	24	10	14	
IV	22	7	15	
**Histologic grade**				***P* = 0.043**
I	17	12	5	
II	54	29	25	
III	15	4	11	
**Lymph node metastasis**				***P* = 0.009**
Negative	45	30	15	
Positive	41	15	26	
**Distant metastasis**				***P* = 0.153**
Negative	63	36	27	
Positive	23	9	14	

### RNF216 downregulation limits CRC cell proliferation and migration

**Figure 2:**
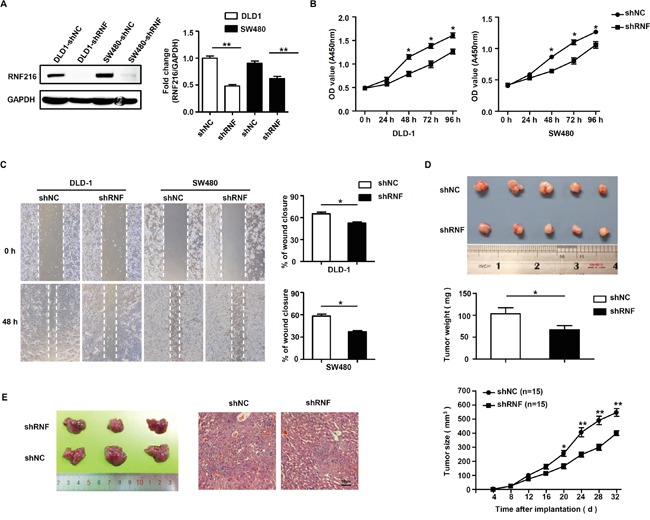
RNF216 promotes CRC cells proliferation and migration *in vitro* and *in vivo* **A.** DLD-1 and SW480 cells were transfected with control (shNC) or *RNF216*-specific shRNA (shRNF). *RNF216* knockdown efficiency was confirmed by immunoblotting of the endogenous protein levels. GAPDH was used as loading control. **B.** Cell proliferation and viability were examined at 0, 24, 48, 72 and 96h. **C.** Wound healing assays measured cell migration at 0 and 48h. **D.** Xenograft model in nude mice. Two different sets of nude mice were injected subcutaneously with DLD-1 DLD-1-shRNF216 or DLD-1-shNC cells. Tumors were measured every four days and removed at day 26. **E.** Liver metastasis model in nude mice. Images and H&E staining of liver metastasis in nude mice injected intrasplenically with DLD-1-shRNF or DLD-1-shNC cells at three weeks post-injection. Scale bar = 50μm. Representative images are shown. Data is expressed as the mean ± SEM of three independent experiments (*P<0.05, **P<0.001).

Because RNF216 is frequently upregulated in CRC, we knocked down *RNF216* in DLD-1 and SW480 cells using a short hairpin RNA (shRNF), with scrambled shRNA as control (shNC). Knockdown efficiency was confirmed by immunoblotting (Figure 2A). *RNF216* knockdown decreased proliferation and migration in both DLD-1 and SW480 cells (Figure 2B & 2C).

To investigate the role of RNF216 in promoting CRC progression *in vivo*, we generated subcutaneous xenograft and liver metastasis models in nude mice using DLD-1 cells transfected with shNC and shRNF. In subcutaneous xenograft models, DLD-1-shNC tumors formed earlier and grew faster compared with the knockdown groups (*P*<0.001, Figure 2D). In liver metastasis models, RNF216 knockdown suppressed liver metastasis (Figure 2E). All together, these results showed that RNF216 promoted tumorigenesis, growth and liver metastasis in nude mice.

### RNF216 negatively regulates CRC cell autophagy

**Figure 3:**
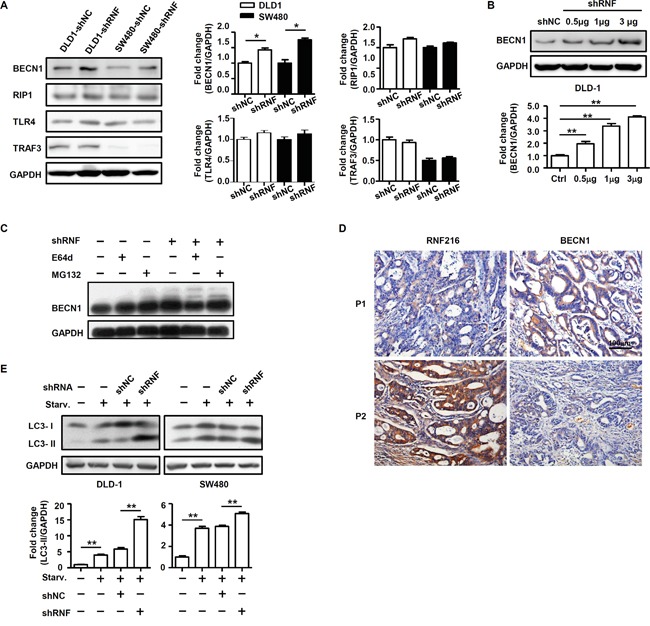
RNF216 promotes proteasomal degradation of BECN1 and negatively regulates CRC cell autophagy **A.** DLD-1 and SW480 cells were transfected with shNC or shRNF. BECN1, RIP1, TLR4 and TRAF3 of DLD-1 and SW480 cells was done by immunoblotting. **B.** BECN1 was detected in DLD-1 cells transiently transfected with shNC, 0.5μg, 1μg and 3μg shRNF by immunoblotting. **C.** DLD-1 cells were treated with E64d or MG132, and BECN1 expression was measured by immunoblotting. **D.** RNF216 and BECN1 IHC staining in serial sections of human CRC. Examples of patients with low and high RNF216 and BECN1 expression are shown. Scale bar = 100μm. **E.** DLD-1 and SW480 cells were subjected to serum starvation for 12h. Autophagy was assessed by immunoblotting for LC3. Data are expressed as the mean ± SEM of three independent experiments (*P<0.05, **P<0.001). GAPDH was used as a loading control.

RNF216 reportedly mediates ubiquitination of multiple molecules, such as TLR4 [18], RIP1 [19], TRAF3 [20], BECN1 [21], and is associated with carcinogenesis. We monitored expression of these molecules in DLD-1 and SW480 cells with or without *RNF216* knockdown. Our results showed no significant changes for TLR4, RIP1 and TRAF3, while BECN1 expression was markedly increased in *RNF216* knockdown cells (Figure 3A & S1). Immunoblotting showed that BECN1 expression was increased as shRNF plasmid volume increased in transfections (Figure 3B), suggesting a strong correlation between RNF216 and BECN1. The proteasome and lysosome are known to be involved in protein degradation. The ubiquitin-proteasome system involves a ubiquitin-activating enzyme, a ubiquitin-conjugating enzyme and a ubiquitin ligase (E3). The E3 ligase determines substrate specificity and most ubiquitinated proteins are then targeted for degradation by the proteasome. We treated DLD-1 cells with MG132, a proteasome inhibitor or E64d, a lysosomal inhibitor. We found that BECN1degradation was blocked by MG132 treatment, while E64d treatment did not affect RNF216-mediated BECN1 degradation (Figure 3C). These results demonstrate that RNF216 promotes BECN1 degradation through the ubiquitin-proteasome pathway.

We then used IHC analysis to examine RNF216 and BECN1 expression in CRC patients. All 86 CRC cases were analyzed and scored for RNF216 and BECN1 expression. Our data showed that high RNF216 level negatively correlated with BECN1 expression (Figure 3D, Table 2), consistent with the role of BECN1 as a tumor suppressor. Because BECN1 plays an essential role in autophagy induction [8, 11, 23–25], we subjected shNC and shRNF-transfected cells to serum starvation (normal medium without serum) and assessed autophagy by monitoring light chain 3 (LC3) via immunoblotting. Starvation increased LC3-II level, and LC3-II was significantly higher in shRNF-compared with shNC-transfected cells, implying that RNF216 restricted CRC cell autophagy during starvation (Figure 3E). Collectively, these results demonstrated that RNF216 was negatively correlated with BECN1 in CRC cells, and *RNF216* knockdown restored autophagy upon starvation.

**Table 2:** The relationship between RNF216 expression and BECN1 in human CRC tissues

		RNF216 expression		Correlation coefficient (R)
		Low	High	
**BECN1**	**Low**	10	20	**R = −0.278 (*P* = 0.0099)**
	**High**	35	21	

### RNF216 induces CRC cell proliferation and migration by inhibiting BECN1-mediated autophagy

**Figure 4:**
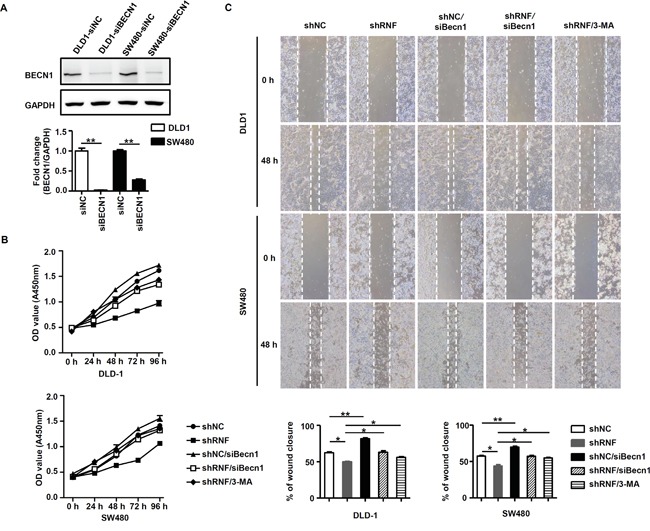
RNF216 induces proliferation and migration via BECN1 ubiquitination and inhibition of autophagy **A.** BECN1 was detected in siNC and siBECN1 transfected DLD-1 and SW480 cells w by immunoblotting. GAPDH was used as loading control. **B.** WST-1 assay was used to detect proliferation and viability in DLD-1 and SW480 cells transfected with shNC, shRNF, siBECN1 or shRNF/siBECN1, or treated with 3-MA (8μM) at 0, 24, 48, 72 and 96h. **C.** Wound healing assay was used to detect migration in DLD-1 and SW480 cells transfected with shNC, shRNF, siBECN1 or shRNF/siBECN1, or treated with 3-MA at 0 and 48h. Data are expressed as the mean ± SEM of three independent experiments (*P<0.05, **P<0.001).

Data from our previous and current studies indicated that RNF216 inhibited autophagy by enhancing BECN1degradation, thus we explored whether RNF216 induced CRC proliferation and migration through this mechanism. *BECN1* was knocked down in DLD-1 and SW480 cells by *BECN1*-specific siRNA or non-specific siRNA as the negative control (Figure 4A). We measured DLD-1 and SW480 cells proliferation and viability following transfection with shNC, shRNF, siBECN1 or shRNF/siBECN1 constructs, with or without 3-MA treatment (an autophagy inhibitor). *RNF216* knockdown decreased proliferation and migration in both cell types compared with controls. In addition, this decrease was almost completely restored by *BECN1* knockdown or 3-MA treatment (Figure 4B & 4C), confirming the role of BECN1 in CRC cell proliferation and migration. These data combined showed that RNF216 promoted CRC cell proliferation and migration by negatively regulating BECN1-dependent autophagy.

## DISCUSSION

RNF216 functions as an E3 ubiquitin-protein ligase and mediates ubiquitination and proteolytic degradation of TLRs and downstream molecules, restricting the intensity and duration of the innate immune response [18–20]. In addition, RNF216 inhibits TLR4-mediated autophagy in macrophages by a TLR4-independent mechanism, i.e. BECN1 ubiquitination [21]. As autophagy has been shown to affect carcinogenesis of multiple cancers, the current study showed that RNF216 is involved in CRC progression, and promotes proliferation and migration.

Increased RNF216 expression in human CRC tissues suggested its role as a potential CRC biomarker. RNF216 expression is positively associated with patients' characteristics, including advanced clinical stage, tumor size and positive lymph node metastasis, highlighting its potential for use in diagnosis and therapeutic guidance.

Our results demonstrated that high RNF216 level promoted CRC cell proliferation and migration, while *RNF216* knockdown inhibited proliferation and migration, both *in vitro* and *in vivo*. Although RNF216 has several interaction partners, such as TLR4, RIP1, TRAF3 and BECN1, our data showed that only BECN1 was significantly upregulated in CRC cells in response to *RNF216* knockdown, supporting that RNF216 contributes to cancer development by regulating autophagy.

Autophagy has dual and contradictory in cancer development [8, 26–29]. Autophagy could promote survival under metabolic stress and cooperatively inhibit a variety of onotherapies, including cytotoxic, targeted therapy and radiotherapy, serving to protect the genome [27, 29–31]. On the other hand, autophagy suppresses CRC in an earlier stage, inhibiting tumorigenesis and growth by downregulating NF-κB signaling, accumulating ROS, increasing DNA damage and other mechanisms [32, 33]. Moreover, monoallelic loss of the essential autophagy gene BECN1 causes susceptibility to metabolic and promotes tumorigenesis [23, 33], which is consistent with our observations that RNF216 could enhance BECN1 degradation dampening autophagy and contributing to cancer cells proliferation. RNF216 upregulation is one of mechanisms leading to CRC cell autophagy inhibition. However, there are currently no commercial compounds or small molecules specific for inhibiting RNF216. And whether there are some other potential targets of RNF216 in CRC development would be interesting.

BECN1 is indispensable for canonical autophagy, regulating autophagic phosphatidylinositol 3-phosphate generation and recruiting additional Atg proteins to orchestrate autophagosome formation [34]. A recent study showed that both BECN1 over- and under-expression could be related to poor colon cancer prognosis, illustrating the multifunctionality of BECN1 and autophagy in cancer aggressiveness [34, 35]. Our results confirmed the inhibitory role of autophagy in CRC development, consistent with other reports of BECN1-mediated tumor progression inhibition.

In summary, our study found that RNF216 expression is increased in CRC tissues and correlates with clinical stage, tumor size and positive lymph node metastasis. We showed that RNF216 contributes to CRC development by promoting cell proliferation and migration via negative regulation of BECN1-dependent autophagy. Thus, RNF216 is a potential biomarker and therapeutic target for inhibiting CRC development and progression.

## MATERIALS AND METHODS

### Patients and specimens

This study was approved by the Biomedical Research Ethics Committee of Shanghai Institutes for Biological Sciences, Chinese Academy of Sciences. All patients provided full consent for the present study. CRC samples were collected from 86 patients undergoing curative-intent surgery at the Department of Surgery, Renji Hospital, Shanghai Jiao Tong University School of Medicine between 2008 and 2011. Additionally, 86 normal colorectal tissues adjacent to colorectal cancer tissues were collected. None of the patients had received any preoperative treatment. All specimens were reviewed by two clinical pathologists to verify histologic diagnoses. Cancers were staged according to the classification of the American Joint Committee on Cancer pathologic tumor-node-metastasis (TNM).

### Cell culture

Human colorectal carcinoma cell lines (HT-29, DLD-1, Colo205, Caco-2, SW480 and SW620) and one normal colon epithelial cell line (CCD-18Co) used in this study were purchased from the American type culture collection (ATCC, Manassas, VA, USA). Cells were cultured in RPMI 1640, DMEM or L-15 (Gibco, Grand Island, NY, USA) medium supplemented with 10% fetal bovine serum, 100U/mL penicillin and 100 mg/mL streptomycin (Gibco, Grand Island, NY, USA) in humidified air at 37°C with 5% CO2.

### Immunohistochemistry assay and the quantification

Sections were subjected to deparaffination, rehydration, antigen retrieval and blockade of nonspecific bindings. The specimens were incubated with primary anti-human RNF216 (Abcam, Cambridge, UK) and BECN1 (Cell Signaling Technology, Danvers, MA, USA) antibodies and second antibody, and then counterstained with hematoxylin and mounted. Immunostaining was independently examined by two clinical pathologists who were unaware of the patient outcome. For each sample, five fields (100×) were randomly selected. Staining intensity and percentage of positive tumor cells were assessed. Staining was quantified as described previously [22].

### Lentiviral vector construction and transfection

The following oligonucleotides were inserted into the lentiviral vector, pLVX-shRNA2, RNF216 (shRNA): 5′-GGACACTATGCAATCACCCG-3′ and scrambled control (shNC): 5′-AGGACTGAGTGTACCGTCT-3′ (Genepharma, Shanghai, China). High titer lentiviral stocks were produced to infect CRC cells, and transfection efficiency exceeded 95%.

### siRNA transfection

Cells were cultured for 24 h prior to transient transfection with BECN1 siRNA, RNF216 siRNA or negative control siRNA (Life Technologies, Grand Island, NY, USA) using Lipofectamine 3000 Transfection Reagent (Invitrogen, Carlsbad, CA, USA) according to the manufacturer's instructions. After 48 h, transfected cells were harvested for immunoblotting and subsequent assays.

### Immunoblotting

Cell lysates were subjected to SDS-PAGE and transferred to polyvinylidene difluoride membranes. Target proteins were detected with primary antibodies against LC3 (Cell Signaling Technology, Danvers, MA, USA), BECN1 (Santa Cruz Biotechnology, Santa Cruz, CA, USA), RNF216 (Abcam, Cambridge, UK), HRP-conjugated anti-mouse and anti-rabbit secondary antibodies (Jackson ImmunoResearch, West Grove, PA, USA). Bands were visualized using Chemilucent Plus Western Blot Kit (Millipore, Darmstadt, Germany). Bands densitometry was quantified by ImageJ software (National Institutes of Health, Bethesda, MD, USA).

### Cell proliferation assay

DLD-1 and SW480 cell lines were seeded in 96-well plate at 3000 cells/well in 100μL medium. WST-1 assay (Roche, Basel, Switzerland) was used to measure viable proliferating cells at 0, 24, 48, 72 and 96 h after plating. Absorbance was measured at 450 nm using a spectrophotometer.

### Wound healing assay

DLD-1 and SW480 cell lines were seeded in 6-well plates at >90% confluency. A wound was created with a sterile 200μL pipet tip. Cells were rinsed with PBS and 1.5 mL of low serum medium was added to the wells. Pictures were taken at 0, 6, 12, 24 and 48 h.

### Animal study

Six-week old BALB/c nude mice were used and treated under specific pathogen-free conditions. To establish CRC xenografts, mice were injected subcutaneously with 5×10^6^ DLD-1 cells in PBS (n=15 per group). Mice were examined every four days and scored according to tumor width and length. Tumor volume was estimated by the formula width^2^ × length × 0.52 in mm^3^. Tumors were removed on day 26. To establish a liver metastasis model, control and *RNF216* knockdown cells were injected into the spleen (5×10^6^ per mouse) of nude mice (n=15 per group). Mice were euthanized and livers were removed on day 21. H&E staining was performed on paraffin embedded liver sections. All animal procedures were approved by the Animal Welfare & Ethics Committee of Shanghai Jiao Tong University School of Medicine.

### Statistical analysis

Differences were evaluated using Statistical Package for Social Science software (version 20.0, SPSS Inc., Chicago, USA). Significant differences were evaluated using an independent-samples *t* test or Wilcoxon rank test, except that multiple treatment groups were compared within individual experiments by ANOVA. The association of staining intensity with clinicopathologic patterns was assessed with Chi-square test and two-sided Fisher's exact test to assess significant differences. Correlation between RNF216 and BECN1 expression were analyzed via Chi-square test. *P*<0.05 was considered significant. All values were presented as mean ±S.E.M.

## SUPPLEMENTARY FIGURE


