# Supplementary material for: RNF216 contributes to proliferation and migration of colorectal cancer via suppressing BECN1-dependent autophagy
Source: Oncotarget. 2016 May 18;7(32):51174–83. doi: 10.18632/oncotarget.9433 (PMC5239467; doi:10.18632/oncotarget.9433)
Supplement: Supplementary file 1 [file oncotarget-07-51174-s001.pdf]

RNF216 contributes to proliferation and migration of colorectal cancer via suppressing BECN1-dependent autophagy

SUPPLEMENTARY FIGURE

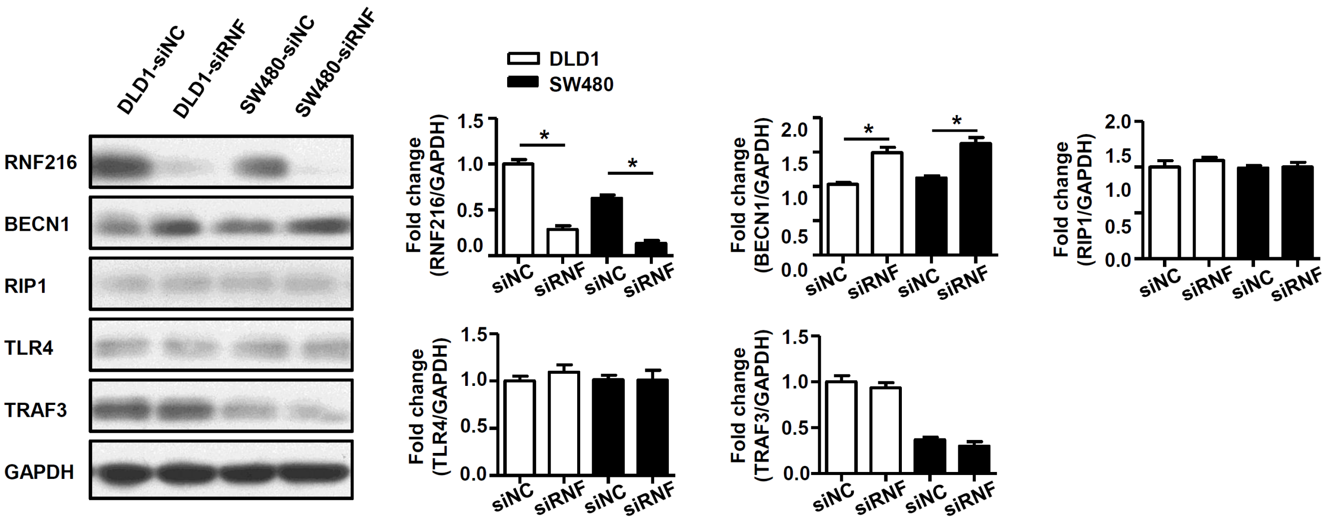

Supplementary Figure S1: DLD-1 and SW480 cells were transiently transfected with siNC and siRNF. RNF216, BECN1, RIP1, TLR4 and TRAF3 were detected in DLD-1 and SW480 cells by immunoblotting. GAPDH was used as loading control. Data are expressed as the mean ± SEM of three independent experiments (**P*<0.05, ***P*<0.001).
